# Life activities of elderly patients with operated nonfunctional pituitary adenoma

**DOI:** 10.3389/fendo.2026.1730144

**Published:** 2026-03-13

**Authors:** Zehra Kara, Suna Avci, Seçil Erden Melikoglu, Cem Sulu, Ahmet Numan Demir, Serhat Uysal, Hande Mefkure Özkaya, Pınar Kadıoğlu

**Affiliations:** 1 Cerrahpasa Medical Faculty, Department of Endocrinology, Metabolism and Diabetes, University of Istanbul-Cerrahpasa, Istanbul, Türkiye; 2 Cerrahpasa Medical Faculty, Department of Geriatrics, University of Istanbul-Cerrahpasa, Istanbul, Türkiye; 3 Department of Fundamentals of Nursing, Florence Nightingale Faculty of Nursing, Istanbul University-Cerrahpasa, Istanbul, Türkiye

**Keywords:** cognition, elderly population, fragility, non-function pituitary adenoma, sarcopenia

## Abstract

**Aim:**

To evaluate the clinical findings of nonfunctional pituitary adenomas in the geriatric population, functional status, cognitive function, quality of life, frailty, and incidence of anxiety and depression during treatment and follow-up.

**Material-method:**

We evaluated operated nonfunctional pituitary adenoma (NFA) cases aged 65 years and older followed up in our clinic between 2010 and 2022. Patients without pituitary adenoma who were followed up in the geriatrics outpatient clinic were included as the control group in the study. Data on clinical, endocrinological, pathological, and radiological findings, as well as on treatment methods, were taken from the files. Bioimpedance was used to assess muscle strength, walking speed, and muscle to fat ratio in patients. In addition, quality of life, anxiety, depression, cognitive function, and vulnerability were assessed.

**Results:**

The study included 43 elderly patients diagnosed with NFA and 60 elderly patients without NFA. The mean ages, sex ratios, and body mass indices (BMI) of patients diagnosed with NFA and the control group were similar (age: 70.9 ± 0.66; 73.1 ± 0.8, p = 0.6; female/male: 24/19, 35/25, p = 0.1; BMI: 28.3 ± 3.9/28.5 ± 4.8, p = 0.9, respectively). Cognition status (MMT: 28;29, p=0.002), frailty scores (2[2-3]; 1[1-2], M[IQR], p<0.001), sarcopenia rates (62%; 30%, p=0.007) was worse in patients with NFA.

**Conclusion:**

In the study comparing patients diagnosed with surgical pituitary NFA with patients of similar age and comorbidity, the frailty score and rate of sarcopenia were higher and their cognition was worse. The findings indicate that complications from surgery and postoperative treatment in patients with pituitary NFA make elderly patients more vulnerable. We emphasize that patients with NFA in this age group should be examined more carefully.

## Introduction

With the developments in modern medicine, the life expectancy of the population is increasing. It is expected that the number of adults over 65 years of age will reach 2 billion by 2050, representing 20% of the world's population (1, 2). The assessment and treatment of medical problems in this age group is therefore becoming increasingly important.

Clinical series have shown that the majority of pituitary adenomas are nonfunctional in patients over 70 years of age. Studies conducted by endocrinologists and neurosurgeons have shown that comorbidities and postoperative complications of nonfunctioning pituitary adenomas are more common in the elderly than in adults. The most common clinical manifestations in NFAs in the elderly are visual disturbances and hypopituitarism. In the postoperative period, diabetes insipidus and continuous vision loss occurred more frequently in the elderly than in adults (3, 4). A study by Crouzeix et al. assessed cognition and quality of life in adult patients with pituitary NFA and found that quality of life did not change and cognitive function was impaired (5).

Studies in the literature on pituitary adenomas in elderly individuals have focused on assessments of compression symptoms and endocrinological functions. No studies have examined the overall functionality of elderly individuals with pituitary adenomas in the postoperative period. In this study, we aimed to evaluate cognitive function, body composition, presence of sarcopenia, quality of life, frailty, anxiety, and depression to predict the follow-up of elderly patients who underwent surgery for pituitary adenomas.

## Material-method

This retrospective study was conducted in a central tertiary university hospital. The study was approved by the Ethics Committee of Istanbul University-Cerrahpaşa, Cerrahpaşa Faculty of Medicine. The study is in full compliance with the Declaration of Helsinki. Patient data were coded and stored anonymously.

We evaluated operated pituitary nonfunctional adenoma (NFA) cases aged 65 years and older followed up in Istanbul University Cerrahpasa, Cerrahpasa Medical Faculty, Endocrinology and Metabolism clinic between 2010 and 2022. Patients without pituitary adenoma who were followed up in the geriatrics outpatient clinic were included as the control group in the study. Clinical, endocrinologic, pathologic, and radiologic findings and treatment information were obtained from the records. Bioimpedance was used to determine muscle strength, walking speed, and muscle-to-fat ratio in patients. In addition, quality of life, anxiety, depression, cognitive function, and vulnerability were assessed.

Inclusion criteria were (I) patients aged 65 years and older, (II) with operated non-functional pituitary adenoma, (III) who can walk.

The exclusion criteria were as follows: (I) dementia, (II) delirium, (III) Parkinson's disease, (IV) ischemic and cerebrovascular vascular disease, (V) psychiatric patients, (VI) patients who cannot walk, (VII) patients under 65 years of age, (X) those with oncology, hematology and rheumatology disease, (XIX) spinal disc herniation, (χ) those with limited mobility due to arthrosis.

### Radiological evaluation

All neuroimaging was performed in our institute using a 3T Philips Ingenia MR scanner (Philips, Best, The Netherlands) with a 16-channel head coil. The imaging protocol included coronal T1-weighted turbo spin-echo images [repetition time (TR), 300 ms; echo time (TE), 10 ms; field of view (FOV), 190x190 mm; slice thickness, 2,5 mm; matrix, 192x192), sagittal T1-weighted turbo spin-echo [repetition time (TR), 350 ms; echo time (TE), 10 ms; field of view (FOV), 190x190 mm; slice thickness, 2,5 mm; matrix, 224x320) and coronal T2-weighted turbo spin-echo (TR, 3090 ms; TE, 104 ms; FOV, 180x180 mm; slice thickness, 2,5 mm; matrix, 224x320). Dynamic contrast-enhanced coronal T1-weighted turbo field echo images (TR, 448 ms; TE, 10 ms; FOV, 190x190 mm; slice thickness, 3 mm; matrix, 224x320) were acquired 0 to 3 min after 0.1 mmol/kg of gadolinium contrast agent (Dotarem; Guerbert, SA) was fully injected at rate of 2 mL/s via an antecubital venous access.

### Walking speed

Physical performance was measured with the 4-meter walking speed test. The threshold for walking speed is < 0.8 m/s (6).

### Evaluation of malnutrition

Mini-nutrition test (MNA) was used to evaluate the nutritional status of the patients. If the value obtained from the MNA short form is 11 and below, the MNA long form is applied. The score obtained from this form is also: 23.5 and above are not malnutrition, 17-23.5 are considered malnutrition risk, 17 points and below are considered malnutrition (7).

### Muscle strength assessment

A Jamar hand dynamometer with a gripping arm was used to determine the patient's grip strength. To determine the dominant hand, participants are asked which hand they use when eating or writing. During the measurements, participants are asked to face forward while seated, with their feet shoulder-width apart and elbows fully extended. The dynamometer is held in a comfortable grip position with the index finger flexed 90 degrees. Patients are asked to apply full force to the arm for 3 seconds. They are told not to hold their breath or shake the force meter during the test. The grip strength of the patient's dominant hand is measured three times, and the highest value is reported in kilograms (kg). There should be a pause of at least 60 seconds between each test. According to the European Working Group on Sarcopenia in the Elderly, the threshold for sarcopenia at < is 20 kg in women and <is 30 kg in men (8).

### Sarcopenia assessment

In order to determine the muscle mass and body fat ratio, the patient was evaluated with the Bodystat quadscan 4000 brand bioimpedance device after 4 hours of fasting. In measurements made with bioimpedance, there is a reference range for muscle and fat mass according to gender and age range (9).

### Examination of fragility

To determine the fragility of the patients, the Fried test questionnaire, valid and reliable in our country, was performed. Fried test: 1. 5-metre walking speed test (slow > 6 sec, very slow > 7.7 sec, forward slow > 10 sec., 2. Grip strength test: female < 20 kg, male < 30 kg, 3: No physical activity in the past year, mostly sedentary or infrequent short walking, 4-Weakness: feeling of difficulty in all daily activities on at least 3 days in the past week or inability to start activities, 5-Weight loss: involuntary weight loss in the past year > 4.5 kg The presence of 3 or more of the criteria is defined as frailty syndrome. The presence of 1 and 2 is interpreted as predisposition to frailty (10).

### Examination of cognition

A minimental test was performed to evaluate the patients' cognitive performance. The test is scored at 30 points. Those with a normal score of 24-30, mild dementia of 18-23, moderate dementia of 17-13, and a score of 12 and below are classified as advanced dementia (11).

### Quality of life evaluation

The general quality of life scale EQ -5D-5L was used to assess quality of life. It consists of two parts; Chapter 1 defines the health profile in five dimensions: mobility, self-care, social life, pain, and psychological state. According to each dimension grade; It contains 5 statements as 0 no problem, 1 mild problem, 2 moderate problems, 4 severe problems, 5 extreme problems. Section 2 evaluates their current state of health on a scale of 0 to 100 (12).

### Depression and anxiety evaluation

The geriatric depression scale includes 30 questions; 10 and below is interpreted as normal, 11–13 points as probable depression, and 14–30 points as definite depression.

The Beck anxiety scale was used to assess patient anxiety. Consisting of 21 questions and each question has never been answered 0 points, mildly but it did not affect me 1 point, it affected me moderately, it was not pleasant but I could bear it 2 points and it affected seriously, I had a hard time enduring it as 3 points interpreted. 0–7 points as no anxiety, 8–15 points as mild anxiety, 16–25 points as moderate anxiety, 26–63 points as severe anxiety (13).

### Statistical analysis

Statistical analyzes were carry out with the Statistical Package for the Social Sciences (SPSS) software (version 21.0). Data were evaluated for normality with the Kolmogorov-Smirnov test. Continuous variables were expressed as mean ± standard deviation (SD) and/or median (interquartile range [IQR]). Student's t test was used when comparing groups with normal data distribution. Medians were compared with the Mann-Whitney U test and the Kruskal-Wallis test. Correlation between variables according to the distribution of the data was calculated using Spearman and Pearson tests. The results were evaluated at the 95% confidence interval. P value < 0.05 was considered statistically significant.

### Sample size

To the difference between two independent means with a significance level 5%, a power of 80%, and an effect size of 0.5, we used the G-power program and found that the required sample size is 102.

## Results

The study included 43 elderly patients diagnosed with NFA by surgery and 60 elderly individuals without NFA. Clinical and demographic characteristics of the groups are shown in **Table 1**, physical examination findings shown in **Tables 2** and laboratory results in **Table 3**.

**Table 1:** Demographic, clinic characteristics of participants .

	*Nonfunctional pituitary adenoma (n=43)*	*Control group (n=60)*	*p*
Age (year), Mean±SD	70.9±0.66	73.1±0.8	0.6
Sex (M/FM), n	19/24	25/35	0.1
Comorbidities			
Hypertension, n (%)	24 (55)	35 (58)	0.2
Type 2 DM, n (%)	9 (21)	16 (26)	0.3
Hyperlipidemia, n (%)	12 (28)	10 (16)	0.6
Benign prostatic hyperplasia, n (%)	6 (14)	12 (20)	0.3
Drugs			
ACE-I/ARB, n (%)	10 (23)	8 (13)	0.2
CCB, n (%)	5 (11)	10 (16)	0.4
α-Blocker, n (%)	6 (14)	12 (20)	0.4
β-Blocker, n (%)	4 (9)	8 (13)	0.5
Metformin, n (%)	8 (18)	13 (21)	0.4
DPP4-I, n (%)	4 (9)	7 (11)	0.5
Statin, n (%)	12 (28)	10 (16)	0.6
Number of drugs used, M[IQR]	4 [2-5]	1 [1-4]	0.006

M, male, FM, Female, DM, Diabetes mellitus, ACE-I, Angiotensin converting enzyme inhibitor, ARB, Angiotensin receptor blocker, CCB, Calcium channel blocker, DPP4-I, Dipeptidyl peptidase 4 inhibitor.

When the data is normally distributed, mean + standard deviation is used..

p <0.05 suggested statistical significance

**Table 2:** Physical examination findings of participants .

	*Nonfunctional pituitary adenoma (n=43)*	*Control group (n=60)*	*p*
BMI, Mean±SD	28.3±3.9	28.5±4.8	0.9
WC/HC, Mean±SD	0.95±0.05	0.94±0.08	0.2
Arm circumference (cm), Mean±SD	29±3.4	29.6±3.3	0.4
Calf circumference (cm), M[IQR]	36 [33-39]	36 [34-39]	0.9
Walking speed (m/sn), M[IQR]	1 [0.87-1.3]	0.68 [0.62-0.85]	<0.001
Muscle strength value (kg) Mean±SD	21.3±6.5	24±7	0.046
Presence of sarcopenia, n (%)	27(62)	18(30)	0.007
Muscle mass (kg), M[IQR]	4.2 [3.1-7.6]	5.4 [4.1-8.3]	0.03
Fat mass (kg), M[IQR]	23 [18-27]	22 [20-25]	0.3
Fragility scale score, M[IQR]	2 [2-3]	1 [1-2]	<0.001
QLS score, M[IQR]	62 [50-75]	65 [55-75]	0.4
GDS score, M[IQR]	11 [5-15]	8 [5-13]	0.7
BAS score , M[IQR]	8 [5-9]	8 [5-12]	0.2
MMT score, M[IQR]	28 [25-29]	29 [27-30]	0.002
MNA score, Mean±SD	27±7.2	26±9.3	0.9

BMI, Body mean index, WC, Waist circumference, HC, Hip circumference, QLS, Quality of Life Scale Score, GDS, Geriatric Depression Score, BAS, Beck Anxiety Scale, MMT, Mini Mental Test, MNA, Mini Nutritional Assessment.

**Table 3:** Laboratuary characteristics of participants.

	*Nonfunctional pituitary adenoma (n=43)*	*Control group (n=60)*	*p*
WBC (103/µ) M[IQR]	4.2 [4.1-4.5]	4.5 [4.4-4.7]	0.8
Hb (g/dl) Mean±SD	11.7±2.3	11±3.1	0.8
Platelet (103/µL) M[IQR]	273 [245-320]	210 [205-241]	0.2
Urea (mg/dL) Mean±SD	30±2.3	28±3.4	0.7
Creatinine (mg/dL) Mean±SD	1.1±0.7	1±0.3	0.8
Na (mEq/L) Mean±SD	136±9	140±11	0.6
K (mEq/L) Mean±SD	4±0.3	4.2±0.2	0.7
Ca (mg/dL) Mean±SD	8.8±0.7	8.6±0.3	0.4
P (mg/dL) Mean±SD	2.3±0.2	2.9±0.2	0.5
Total protein (g/dl) M[IQR]	7[6.7-.7.5]	6.4 [6.2-6.8]	0.1
Albumin (g/dl) M [IQR]	4.1 [4-4.3]	3.6 [3.4-4]	0.2
AST (IU/L) M[IQR]	14 [12-17]	15 [14-20]	0.1
ALT (IU/L) M[IQR]	11 [10-18]	17 [14-23]	0.2
Cortisol (µg/dl) Mean±SD	12±2	13±4.5	0.8
ACTH (pg/ml) M[IQR]	15 [11-19]	12 [10-28]	0.2
TSH (µIU/ml) Mean±SD	1.5±0.3	2±0.7	0.1
freeT4 (ng/dl) Mean±SD	1.2±0.2	1.4±0.1	0.3
freeT3 (pg/ml) Mean±SD	2.2±0.2	2.7±0.1	0.2
GH (µg/L) M[IQR]	0.5 [0.1-1]	0.5 [0.2-1.2]	0.9
IGF-1 (ng/ml) M[IQR]	114 [98-143]	121 [100-138]	0.2
LH (mIU/ml) M[IQR]	24±7.8	29±5	0.1
FSH (mIU/ml) Mean±SD	26±4.4	31±5.2	0.2
Estradiol (pg/ml) Mean±SD	16±1.3	20±3	0.1
Testesterone (ng/dl) M[IQR]	210 [187-267]	235[190-296]	0.3
Prolactin (µg/L) Mean±SD	17±3.2	16±7	0.7
25(OH) Vitamin D (µg/L) Mean±SD	41±13	36±15	0.4
Vitamin B12 (pg/ml) Mean±SD	486±84	455±92	0.8

WBC, White blood cell, Hb, Hemoglobin, Na, Sodium, K: Potassium, Ca, Calcium, P, Phosphorus, AST, Aspartate aminotransferase, ALT, Alanine aminotransferase, ACTH, Adrenocorticotropic hormone, TSH, Thyroid stimulating hormone, GH, Growth hormone, IGF-1, Insulin-like growth factor, LH, Luteinizing hormone, FSH, Follicle stimulating hormone, PTH, Parathormone, HbA1c, Glycosylated hemoglobin, M: Median, IQR: Interquartile range.

When the data is normally distributed, mean + standard deviation is used.. When the data is not normally distributed, the median and interquartile range is used. p <0.05 suggested statistical significance

The number of macroadenomas in NFAs was 36 (83%). Patients with NFA presented with headache (60%), hypopituitarism (41%), visual disturbances (15%), and diabetes insipidus (11%). The median follow-up time at NFA was 52 [IQR, 40-96] months. Of the 43 patients with NFA, 6 underwent transsphenoidal surgery twice and the remaining once at the pituitary gland. Ten patients (23%) had residual tumors. The average residual tumor size of the patients was 4.5 ± 1.2 mm. The time elapsed after the surgery of the patients was 49 [IQR, 40-78] months. Postoperative gamma-knife radiotherapy was performed in 2 patients with NFA. Two patients received radiotherapy 4 and 7 years ago.

In the postoperative period, hypopituitarism occurred in 14 patients (32%) during follow-up. Because of central pituitary insufficiency, 11 patients received hydrocortisone, 10 patients received L-thyroxine, and 1 patient received desmopressin and 1 patient received testosterone. Patients with pituitary-derived cortisol deficiency were taking a mean steroid dose equivalent to 23.1 ± 8.1 mg hydrocortisone. The polypharmacy rate of patients in the NFA group was 32% (n=14) and 8% (n=5) in the control group (p=0.048).

### Chronic diseases

In the group with pituitary NFA, 24 patients had hypertension, 9 patients had type 2 diabetes mellitus, 12 patients had hyperlipidemia, and 6 patients had benign prostatic hyperplasia. Patients who received care in the geriatric outpatient clinic formed the control group. In this patient group, 35 patients were diagnosed with hypertension, 16 with type 2 diabetes mellitus, 10 with hyperlipidemia, and 12 with benign prostatic hyperplasia.

Blood glucose levels of diabetics in both groups were adjusted by oral antidiabetic drugs. HbA1c values were similar in the NFA group and the control group (6.9 ± 1.4 and 7.2 ± 1.2, respectively (p=0.4)). No microvascular or macrovascular complications occurred in the diabetic patients in the study.

### Frailty, sarcopenia and cognition

In the assessment of frailty according to the Fried test: 13 (30%) of the patients with pituitary NFA were classified as frail with 3 points, 21 (49%) with 2 points, and 9 (21%) with 1 point as frailty predisposition. In the control group, 8 (13%) were evaluated as not frail with a score of 0, 30 (50%) with a score of 1, and 22 (37%) with a score of 2 as prone to frailty. One of the components of frailty was muscle strength, 21 kg in the NFAs versus 24 kg in the control group (p=0.04), The other component, walking speed, was low at 1 meter/second in the pituitary NFA group and normal at 0.68 meter/second in the control group (p<0.001).

BMI and fat mass of the two groups were similar, but muscle mass was 4.2 and 5.4 kg in the NFA and control groups, respectively (p=0.03). In parallel with this result, sarcopenia rates were higher in patients with NFA than in control patients (62% and 30%, respectively, p=0.007).

The two groups with similar GDS and BAS scores, and their cognitions according to MMT, had lower scores in patients with pituitary NFA compared to control patients (28;29, p=0.002, respectively).

### Correlation analysis

**Figure 1:**
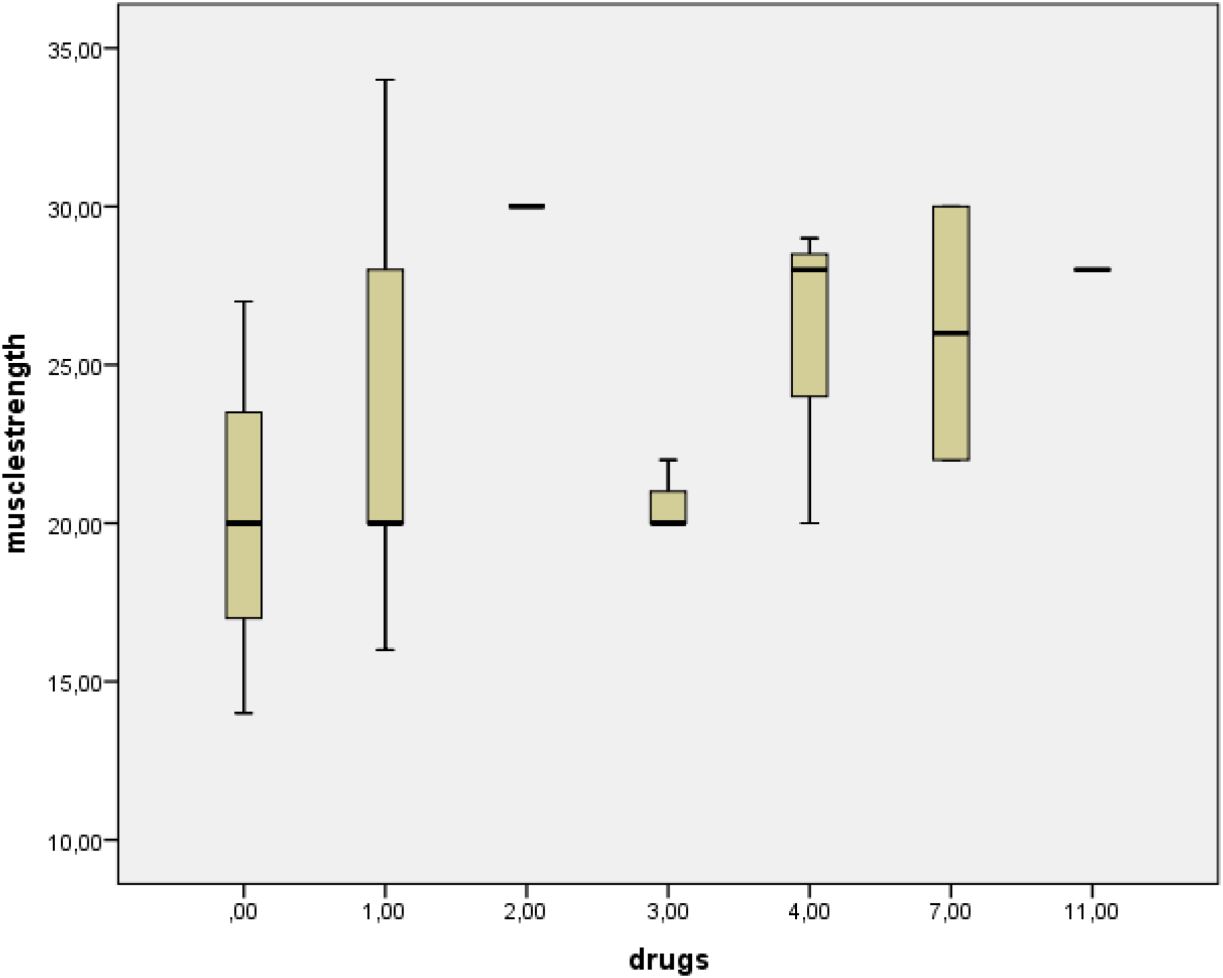
Relationship between the number drugs and muscle strength.

A weak correlation was found between the glucocorticoid dose used and the frailty score and cognition. There was also a relationship between muscle strength and the number of medications taken (**Figure 1**). Correlation analysis results are given in **Table 4**.

**Table 4:** Correlation analysis.

Fried score	Sarcopenia	r=0.312	p=0.015
	Walking speed	r=-0.603	p<0.001
	QLS score	r=0.486	p<0.001
	Glucocorticoid dose	r=0.321	p=0.004
Muscle strength	GDS	r=-0.330	p=0.004
	BAS	r=-0.413	p<0.001
	QLS score	r=-0.417	p<0.001
	MMT	r=0.347	p<0.001
	Number of drugs	r=-0.383	p=0.05
MMT score	BAS score	r=-0.355	p=0.002
	QLS score	r=-0.304	p=0.008

QLS, Quality of Life Scale Score, GDS, Geriatric Depression Score, BAS, Beck Anxiety Scale, MMT, Mini Mental Test., r: correlation coefficient.

## Discussion

In our study, we found that patients with NFA had higher frailty scores, higher rates of sarcopenia, and poorer cognitive function compared to individuals without NFA.

In a study conducted in 27 elderly patients with nonfunctional pituitary macroadenoma, it was found that 50% of the patients had visual disturbances, 30% had headaches, and memory impairment. Endocrinological examination of patients revealed global anterior hypopituitarism in 33% and partial hypopituitarism in 37% of patients (14). In another study, 48 elderly patients with a nonfunctioning pituitary adenoma were studied. Their study found a macroadenoma rate of 92%, extrasellar extension of 88%, and postoperative hypopituitarism of 67% (15). Similarly, in our study, the majority of patients had macroadenoma. They presented with compression symptoms such as headache, hypopituitarism, and visual defects. With these results, we assumed that hypopituitarism may be overlooked in elderly patients.

Frailty is a clinical condition characterized by decreased tolerance to stressors due to diminished homeostatic reserves. The prevalence of frailty increases with age, female sex, depression, malnutrition, and prevalence of chronic diseases (16). The endocrine system is thought to influence fragility due to the complex relationship between the brain, immune system, and skeletal muscle. The hypothalamo-pituitary axis has a key role in fragility (17). Glucocorticoid secretion, androgen production and insulin-like growth factor explain this situation. Since the main components of fragility are muscle mass and muscle strength, activities of daily living and level of movement are adversely affected (18). In this study, vitamin D level was sufficient in both groups and malnutrition was not detected. In our study, it was seen that the NFA group, central adrenal and thyroid hormone insufficiency, and steroid use negatively affected the level of muscle mass and walking speed, which resulted in more fragility.

Endocrine function declines with age because many hormones decrease with age and hormone receptors become less sensitive. A decrease in hormones important for muscle maintenance such as insulin-like growth factor-1 (IGF-1), testosterone, estrogen and dehydroepiandrosterone (DHEA) contributes to sarcopenia (19). In our study, one third of the control group was sarcopenic, which is a significant proportion. It is known that the frequency of sarcopenia increases with age. In our study, we found that the rate of sarcopenia was higher in the study group than in the control group. Although the patients received GC replacement doses, we believe this was an effective factor for sarcopenia. One study examined risk factors for the development of sarcopenia in patients with rheumatoid arthritis. Taking 3.25 mg/day of glucocorticoids for more than a year was found to be a risk factor (20).

Another factor affecting sarcopenia is medications. A meta-analysis of 29 studies reviewed found a higher prevalence of polypharmacy in individuals with sarcopenia. Increasing concern about clinical management of comorbidities that increase with age leads to inappropriate prescribing of medications (21). It is well known that polypharmacy can lead to adverse side effects regardless of the patient's clinical condition. Oral antidiabetic agents may induce myostatin transcription factors and lead to inadequate response to hypertrophic responses to exercise. Beta-blockers may impair muscle adaptation to exercise. Glucocorticoids reduce the expression of mediator molecules involved in mitochondrial functions (22). In this study, it was found that the rate of sarcopenia was higher in the NFA study group than in the control group, possibly due to a higher rate of polypharmacy.

Cognitive impairment in memory and executive functions has been reported in pituitary adenomas. This is attributed to hormonal imbalance and damage to the neuroanatomical structures required for normal memory processing (23). The cognitions of patients with NFA with a mean age of 62 years and undergoing transsphenoidal surgery an average of 8.5 years ago were evaluated. In their study, patients with NFA were compared with the reference population and they found that their cognition was worse. They found that RT had no effect on cognition (24). In another study, patients with NFA underwent neuropsychological assessment before and after transsphenoidal surgery. Patients with NFA performed worse than the control group in 6 of 7 cognitive domains (25). In a study evaluating cognition and quality of life in adult patients with pituitary NFA, cognitive function was found to be impaired but quality of life did not change (5). In our study, the results of the cognitive assessment are consistent with the literature. While age-related cognitive decline is recognized, in our study, the NFA group had lower Mini-Mental Test scores than others in the same age group. However, the results of QLS, GDS, and BAS were similar.

Chen and colleagues found that fragility increases after heart surgery and is associated with prolonged exposure to anesthesia, extended endotracheal tube use, and long intensive care or hospital stays (26). A multicenter study found that in the elderly population, increasing age, longer anesthesia duration, second surgery, and postoperative pulmonary complications were associated with a decline in cognitive function caused by hypoxemia and hypotension (27). In elderly hospitalized patients, prolonged length of stay, repeated hospitalizations, and limited mobility have been associated with an increased frequency of sarcopenia (28). Although we do not know the levels of frailty, cognitive function, or frequency of sarcopenia (19, 21, 25) in our patients before hospitalization, we believe these may have been affected by the procedures performed and the hospital stay.

Current findings indicate that it is difficult to definitively determine whether fragility arises from NFA itself or from surgical and postoperative processes, but both pathways contribute to the fragile phenotype through complementary mechanisms. NFA-related hypothalamo-pituitary axis dysfunction reduces muscle protein synthesis due to chronic deficiencies in IGF-1, gonadal steroids, and thyroid hormones, impairing neuromuscular transmission and mitochondrial capacity and predisposing to sarcopenia (19). Long-term glucocorticoid replacement after surgery activates myostatin and inflammatory pathways that increase muscle catabolism, suppresses the anabolic response to exercise, and exacerbates muscle strength loss (22). Combined with polypharmacy, this further reduces functional reserve through drug-drug interactions and decreased physical activity tolerance (21). Decreased hormone receptor sensitivity with age (19), comorbidities, and immobilization associated with surgery act synergistically with these endocrine and pharmacological effects, resulting in reduced walking speed, cognitive impairment, and decline in daily living activities. Consequently, the biological effects of NFA and chronic factors related to surgery and treatment are not entirely independent, but form a common pathophysiological network that drives frailty and sarcopenia through the gradual depletion of physiological reserves.

### Limitations

This is a cross-sectional study, the evaluation of patients before transsphenoidal surgery would allow us to see the effects of the adenoma itself and the effects of the surgery. It is a study done at a single center and with a small patient group; studies with a large patient group might give healthier results.

### Conclusions

Although many studies have focused on pituitary tumors, research examining NFAs from the perspective of age-related functional decline is limited. The present study found that elderly patients who underwent surgery for NFA were more frail than individuals of similar age and comorbidities, experiencing slower mobility due to muscle wasting and a corresponding decline in cognitive function. These results suggest that this age group should be monitored more closely.

## Data Availability

The raw data supporting the conclusions of this article will be made available by the authors, without undue reservation.

## References

[1] Ezeh AC Bongaarts J Mberu B . Global population trends and policy options. lancet. (2012) 380:142–8. doi: 10.1016/S0140-6736(12)60696-5, PMID: 22784532

[2] United Nations, Department of Economic and social Affairs, Population Division . World Population Prospects: The 2015 revision, key findings and advance tables. In: Technical report. Working paper no. EsA/P/WP. New York: United Nations (2015). p. 241.

[3] Barker FG Klibanski A Swearingen B . Transsphenoidal surgery for pituitary tumors in the United States, 1996–2000: mortality, morbidity, and the effects of hospital and surgeon volume. J Clin Endocrinol Metab. (2003) 88:4709–19. doi: 10.1210/jc.2003-030461, PMID: 14557445

[4] Freda PU Bruce JN . Risks of pituitary surgery in the elderly. Nat Rev Endocrinol. (2010) 6:606–8. doi: 10.1038/nrendo.2010.170, PMID: 20962868 PMC3677229

[5] Crouzeix G Morello R Thariat J Morera J Joubert M Reznik Y . Quality of Life but not Cognition is Impacted by Radiotherapy in Patients with Non-Functioning Pituitary Adenoma. Horm Metab Res. (2019) 51:178–85. doi: 10.1055/a-0850-9448, PMID: 30861564

[6] Abellan van Kan G Rolland Y Andrieu S Bauer J Beauchet O Bonnefoy M . Gait speed at usual pace as a predictor of adverse outcomes in community-dwelling older people an International Academy on Nutrition and Aging (IANA) Task Force. J Nutrition Health Aging. (2009) 13:881–9. doi: 10.1007/s12603-009-0246-z, PMID: 19924348 PMC12878092

[7] Guigoz Y Lauque S Vellas BJ . Identifying the elderly at risk for malnutrition. Mini Nutr Assess Clin Geriatr Med. (2002) 18:737–57. doi: 10.1016/s0749-0690(02)00059-9, PMID: 12608501

[8] Cruz-Jentoft AJ Baeyens JP Bauer JM Boirie Y Cederholm T Landi F . Sarcopenia: European consensus on definition and diagnosis Report of the European Working Group on Sarcopenia in Older. Age Ageing. (2010) 39:412–23. doi: 10.1093/ageing/afq034, PMID: 20392703 PMC2886201

[9] Chen LK Liu LK Woo J Assantachai P Auyeung TW Bahyah KS . Sarcopenia in Asia: consensus report of the Asian. Working Group for Sarcopenia. . J Am Med Dir Assoc. (2014) 15:95–101. doi: 10.1016/j.jamda.2013.11.025, PMID: 24461239

[10] Dent E Kowal P Hoogendijk EO . Frailty measurement in research and clinical practice: A review. Eur J Intern Med. (2016) 31:3–10. doi: 10.1016/j.ejim.2016.03.007, PMID: 27039014

[11] Norris D Clark MS Shipley S . The mental status examination. Am Fam Phisician. (2016) 94:635–41. 27929229

[12] Thompson AJ Turner AJ . A comparison of the EQ-5D-3L and EQ-5D-5L. Pharmacoeconomics. (202) 38:575–91. doi: 10.1007/s40273-020-00893-8, PMID: 32180162

[13] Shimizu U Aoki H Sakagami M Akazawa K . Walking ability, anxiyete and depression, signicantly decrease EuroQol 5-Dimension 5-Level scores in older hemodialysis patients in Japan. Arch Gerontol Geriatr. (2018) 78:96–100. doi: 10.1016/j.archger.2018.06.006, PMID: 29936330

[14] Del Monte P Foppiani L Ruelle A Andrioli G Bandelloni R Quilici P . Clinically non-functioning pituitary macroadenomas in the elderly. Aging Clin Exp Res. (2007) 19:34–40. doi: 10.1007/BF03325208, PMID: 17332719

[15] Villar-Taibo R Díaz-Ortega C Sifontes-Dubon M Fernández-Pombo A Serramito-García R Martínez-Capoccioni G . Pituitary surgery in elderly patients: a safe and effective procedure. Endocrine. (2021) 72:814–22. doi: 10.1007/s12020-021-02665-6, PMID: 33665774

[16] Clegg A Young J Iliffe S Rikkert MO Rockwood K . Frailty in elderly people. Lancet. (2013) 381:752–62. doi: 10.1016/S0140-6736(12)62167-9, PMID: 23395245 PMC4098658

[17] Clegg A Hassan-Smith Z . Frailty and the endocrine system. Lancet Diabetes Endocrinol. (2018) 6:743–52. doi: 10.1016/S2213-8587(18)30110-4, PMID: 30017798

[18] Kamwa V Welch C Hassan-Smith ZK . The endocrinology of sarcopenia and frailty. Minerva Endocrinol (Torino). (2021) 46:453–68. doi: 10.23736/S2724-6507.20.03198-3, PMID: 33331737

[19] Gupta P Kumar S . Sarcopenia and endocrine ageing: are they related? Cureus. (2022) 14:e28787. doi: 10.7759/cureus.28787, PMID: 36225400 PMC9533189

[20] Yamada Y Tada M Mandai K Hidaka N Inui K Nakamura H . Glucocorticoid use is an independent risk factor for developing sarcopenia in patients with rheumatoid arthritis: from the CHIKARA study. Clin Rheumatol. (2020) 39:1757–64. doi: 10.1007/s10067-020-04929-4, PMID: 31938882

[21] Fielding RA Vellas B Evans WJ Bhasin S Morley JE Newman AB . Sarcopenia: an undiagnosed condition in older adults. Current consensus definition: prevalence, etiology, and consequences. International working group on sarcopenia. J Am Med Dir Assoc. (2011) 12:249–56. doi: 10.1016/j.jamda.2011.01.003, PMID: 21527165 PMC3377163

[22] Prokopidis K Giannos P Reginster JY Bruyere O Petrovic M Cherubini A . Sarcopenia is associated with a greater risk of polypharmacy and number of medications: a systematic review and meta-analysis. J Cachexia Sarcopenia Muscle. (2023) 14:671–83. doi: 10.1002/jcsm.13190, PMID: 36781175 PMC10067503

[23] Chieffo DPR Lino F Ferrarese D Belella D Pepa GMD Doglietto F . Brain tumor at diagnosis: from cognition and behavior to quality of life. Diagnostics (Basel). (2023) 13:541. doi: 10.3390/diagnostics13030541, PMID: 36766646 PMC9914203

[24] Brummelman P Elderson MF Dullaart RP van den Bergh AC Timmer CA van den Berg G . Cognitive functioning in patients treated for nonfunctioning pituitary macroadenoma and the effects of pituitary radiotherapy. Clin Endocrinol (Oxf). (2011) 74:481–7. doi: 10.1111/j.1365-2265.2010.03947.x, PMID: 21133979

[25] Butterbrod E Gehring K Voormolen EH Depauw PRAM Nieuwlaat WA Rutten GJM . Cognitive functioning in patients with nonfunctioning pituitary adenoma before and after endoscopic endonasal transsphenoidal surgery. J Neurosurg. (2019) 133:709–16. doi: 10.3171/2019.5.JNS19595, PMID: 31443073

[26] Chen WY Liu CY Shih CC Chen YS Cheng HW Chiou AF . Factors associated with frailty in patients undergoing cardiac surgery: A longitudinal study. J Cardiovasc Nurs. (2022) 37:204–12. doi: 10.1097/JCN.0000000000000787, PMID: 34145204

[27] Moller JT Cluitmans P Rasmussen LS Houx P Rasmussen H Canet J . Long-term postoperative cognitive dysfunction in the elderly ISPOCD1 study. ISPOCD investigators. International Study of Post-Operative Cognitive Dysfunction. Lancet. (1998) 351:857–61. doi: 10.1016/s0140-6736(97)07382-0, PMID: 9525362

[28] Zhou Y Hellberg M Svensson P Höglund P Clyne N . Sarcopenia and relationships between muscle mass, measured glomerular filtration rate and physical function in patients with chronic kidney disease stages 3-5. Nephrol Dial Transplant. (2018) 33:342–8. doi: 10.1093/ndt/gfw466, PMID: 28340152

